# The complete mitochondrial genome of *Psylliodes balyi* Jacoby (Coleoptera: Chrysomelidae)

**DOI:** 10.1080/23802359.2020.1791752

**Published:** 2020-07-20

**Authors:** Xin-Ju Gao, Heng-Liang Wang, Jun-Huai Zu

**Affiliations:** Henan Key Laboratory of Crop Pest Control, IPM Key Laboratory in Southern Part of North China for Ministry of Agriculture, International Joint Research Laboratory for Crop Protection of Henan, Biological Pesticides Engineering Research Center of Henan Province, Institute of Plant Protection, Henan Academy of Agricultural Sciences, Zhengzhou, China

**Keywords:** *Psylliodes balyi*, Chrysomelidae, mitogenome

## Abstract

The complete mitogenome of *Psylliodes balyi* Jacoby (GenBank accession number MT644112) is 14,561 bp in length, and contains 13 protein-coding genes (PCGs), 22 transfer RNA genes (tRNAs), 2 ribosomal RNA genes, and a putative control region. The gene content and orientation of *P. balyi* were identical to other beetle mitogenomes. ATT, ATA, and ATG were initiation codons and TAA, TAG, and T were termination codons. All the 22 tRNAs have the typical cloverleaf secondary structure, except for *trnS_1_* which lacked the dihydrouracil (DHU) arm. The phylogenetic relationship based on the neighbor-joining method showed that *P. balyi* is closely related to *Agasicles hygrophila*, which agrees with the conventional classification.

The eggplant flea beetle, *Psylliodes balyi* Jacoby (Coleoptera: Chrysomelidae), is a major pest of eggplants and distributed widely in Southern China. The adults of *P. balyi* feed on the leaves and the larvae bore into the roots of eggplants, causing great economic losses in eggplant cultivated areas (Yang et al. 2010). In this study, adult species of *P. balyi* were collected from the vegetable field in Guizhou province of China (N26°33′, E106°46′). The samples were preserved in 95% ethanol and stored in the insect specimen room of Henan Academy of Agricultural Sciences with an accession number HAAS-Col-20190901.

The complete mitochondrial genome of *P. balyi* (GenBank accession number MT644112) is a circular DNA molecule of 14,561 bp in length, and contains 13 protein-coding genes (PCGs), 22 transfer RNA genes (tRNAs), two ribosomal RNA genes (*rrnL* and *rrnS*), and a putative control region. The gene content and orientation of *P. balyi* were identical to other beetle mitogenomes (Li et al. 2016; Zhou et al. 2016). Twenty-three genes were encoded on the minor strand (J-strand), while the others were transcribed on the major strand (N-strand). The overall base composition was as follows: A (34.16%), T (42.15%), C (11.59%), and G (12.10%), with an A + T bias (76.31%). The AT-skew and GC-skew of this genome were −0.105 and 0.021, respectively. Gene overlaps were found at 17 gene junctions and involved a total of 55 bp, and the longest overlap (8 bp) existed between *trnY* and *cox1*. There were four intergenic spacer regions ranging in length from 2 to 17 bp, comprising a total length of 36 bp. The largest intergenic spacer sequence of 17 bp was located between *trnS_2_* and *nad1*. The 22 tRNAs had a total of 1446 bp, and their individual lengths ranged from 63 bp (*trnE* and *trnF*) to 72 bp (*trnW*). Consistent with most animal mitogenomes (Wolstenholme 1992; Yuan et al. 2016), all the 22 tRNAs have the typical cloverleaf secondary structure, except for *trnS_1_* which lacked the dihydrouracil (DHU) arm. The *rrnL* and *rrnS* were 1280 and 742 bp in length, with the A + T contents of 81.88% and 81.67%, respectively. The control region was located between *rrnS* and *trnI* with a length of 17 bp, and the A + T content was 88.24%.

All the 13 PCGs start with typical ATN codons, including two ATAs (*nad3* and *nad4L*), five ATGs (*atp6*, *cob*, *cox2*, *cox3*, and *nad4*), and six ATTs (*atp8*, *cox1*, *nad1*, *nad2*, *nad5*, and *nad6*). Nine PCGs terminate with conventional stop codons (TAA or TAG), and the remaining PCGs including *cox1*, *cox2*, *cox3*, and *nad5* use single T as a stop codon. According to the relative synonymous codon usage analyses of 13 PCGs, TTA (L), TTT (F), ATT (I), and ATA (M) were the four most frequently used codons. Leucine, isoleucine, phenylalanine, and methionine are the most frequent amino acid of 13 PCGs. Based on the concatenated amino acid sequences of 13 PCGs, the neighbor-joining method was used to construct the phylogenetic relationship of *P. balyi* with 15 other beetles. The result showed that *P. balyi* was closely related to *Agasicles hygrophila* (Figure 1), which agrees with the conventional classification.

**Figure 1.**
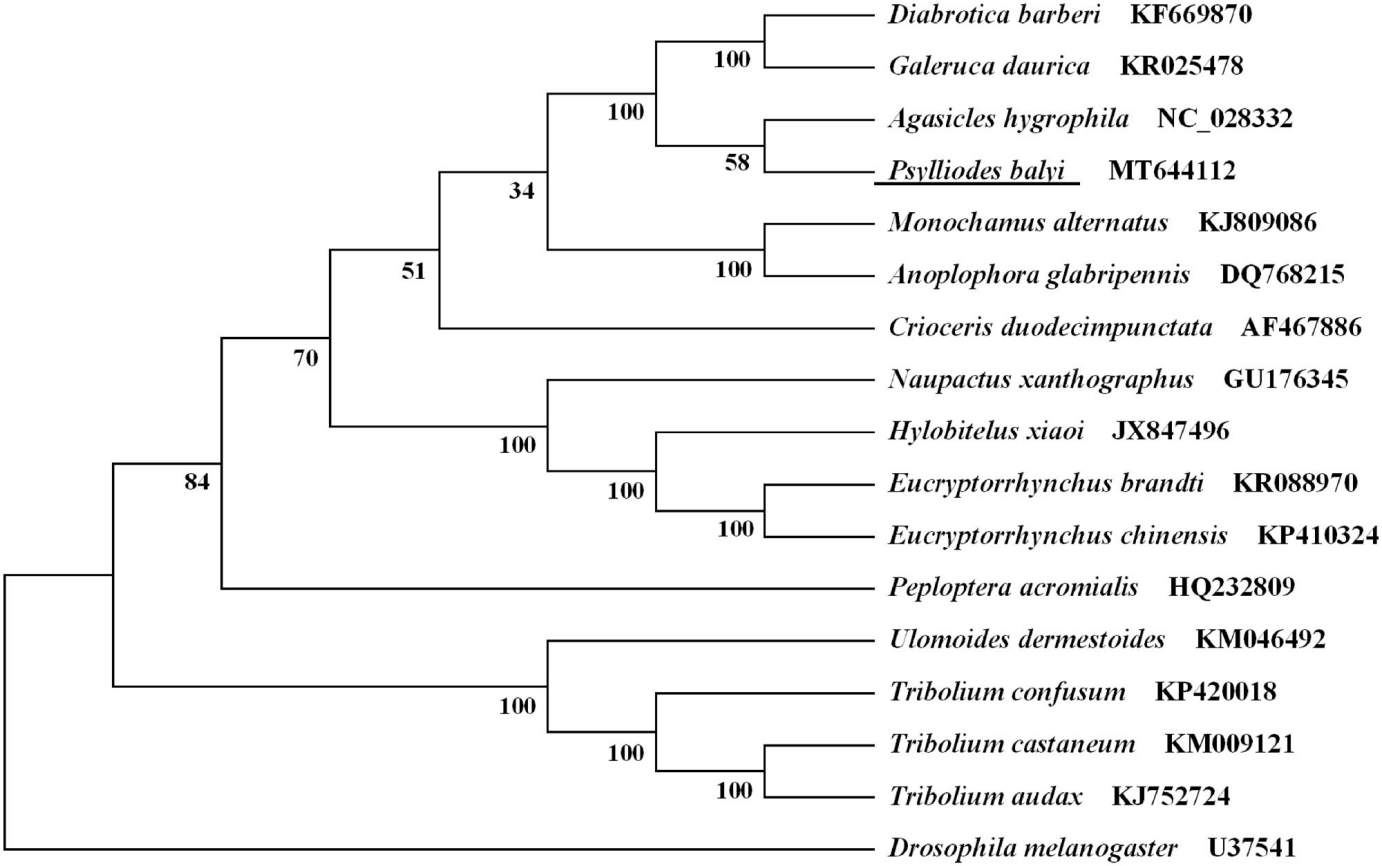
Phylogenetic tree showing the relationship between *Psylliodes balyi* and 15 other beetles based on neighbor-joining method. *Drosophila melanogaster* was used as an outgroup. GenBank accession numbers of each species were listed in the tree. The beetle determined in this study is underlined.

## Data Availability

The data that support the findings of this study are openly available in GenBank at https://www.ncbi.nlm.nih.gov/genbank/, reference number MT644112.
